# The ROCK trial—a multidisciplinary Rehabilitation intervention for sudden Out-of-hospital Cardiac arrest survivors focusing on return-to-worK: a pragmatic randomized controlled trial protocol

**DOI:** 10.1186/s13063-024-07911-6

**Published:** 2024-02-01

**Authors:** Jan Christensen, Bo Gregers Winkel, Lilli Kirkeskov, Fredrik Folke, Matilde Winther-Jensen, Christine Eckhardt-Bentsen, Jesper Kjærgaard, Christian Hassager, Mette Kirstine Wagner

**Affiliations:** 1 Department of Occupational Therapy and Physiotherapy, Copenhagen University Hospital-Rigshospitalet, 2100 Copenhagen, Denmark; 2 Department of Public Health, University of Copenhagen, Copenhagen, Denmark; 3 Department of Cardiology, Copenhagen University Hospital-Rigshospitalet, Copenhagen, Denmark; 4 Center of Social Medicine, Copenhagen University Hospital-Bispebjerg and Frederiksberg, Copenhagen, Denmark; 5 Department of Cardiology, Copenhagen University Hospital-Herlev and Gentofte Hospital, Hellerup, Denmark; 6 Department of Clinical Medicine, University of Copenhagen, Copenhagen, Denmark; 7 Department of Data, Biostatistics and Pharmacoepidemiology, Center for Clinical Research and Prevention, Copenhagen University Hospital-Bispebjerg and Frederiksberg, Copenhagen, Denmark

**Keywords:** Sudden cardiac arrest, Cardiac arrest, Cardiac rehabilitation, Rehabilitation, Return-to-work

## Abstract

**Introduction:**

Most cardiac arrest survivors are classified with mild to moderate cognitive impairment; roughly, 50% experience long-term neurocognitive impairment. Postarrest challenges complicate participation in society and are associated with social issues such as failure to resume social activities and impaired return to work. The effectiveness of rehabilitation interventions for out-of-hospital cardiac arrest survivors are sparsely described, but the body of evidence describes high probabilities of survivors not returning to work, returning to jobs with modified job descriptions, returning to part-time employment, and often in combination with extensive unmet rehabilitation needs. Hence, there is a need to develop and test a pragmatic individual targeted intervention to facilitate return to work (RTW) in survivors of OHCA. The overall aim of the ROCK trial is to evaluate the effectiveness of a comprehensive individually tailored multidisciplinary rehabilitation intervention for survivors of OHCA on RTW compared to usual care.

**Methods and analysis:**

The ROCK trial is a two-arm parallel group multicentre investigator-initiated pragmatic randomized controlled superiority trial with primary endpoint measured 12 months after the cardiac arrest. Adult survivors who were part of the labour force prior to the OCHA and had at least 2 years until they are qualified to receive retirement state pensions are eligible for inclusion. Survivors will be randomized 1:1 to usual care group or usual care plus a comprehensive tailored rehabilitation intervention focusing on supporting RTW. After comprehensive assessment of individual rehabilitation needs, the intervention is ongoingly coordinated within a multidisciplinary rehabilitation team, and the intervention can be delivered for up until 12 months. Data for the primary outcome will be obtained from the national register on social transfer payments. The primary outcome will be analysed using logistic regression assessing RTW status at 12 months adjusting for the intervention and age at OHCA, sex, marital status, and occupation prior to OHCA.

**Discussion:**

The ROCK trial is the first RCT to investigate the effectiveness of a rehabilitation intervention focusing on return to work after cardiac arrest.

**Trial registration:**

ClinicalTrials.gov NCT05173740. Registered on May 2018

**Supplementary Information:**

The online version contains supplementary material available at 10.1186/s13063-024-07911-6.

## Background

Each year, more than 5000 citizens experience an out-of-hospital cardiac arrest (OHCA) in Denmark. In 2021, the Danish 30-day survival rate was 13% equivalent to yearly incidence of 610 OCHA survivors [1].

Rehabilitation targeting physical, neurological, and psychological consequence after OHCA is recommended in international clinical guidelines. Due to hypoxic–ischaemic brain injury, the course of acute post-cardiac arrest care and the trauma of resuscitation the frequency of cognitive impairment and emotional reactions are high in survivors of cardiac arrest. These problems affect survivors and restrict their ability to return-to-work (RTW) [2, 3]. Although the majority of cardiac arrest survivors are classified with mild to moderate cognitive impairment, roughly 50% experience long-term neurocognitive impairment. Thus, most cognitive recovery is found to occur during the first 3 months after resuscitation [4, 5]. Looking at emotional issues, both depression, anxiety, and post-traumatic stress disorder are found to affect approximately 20–25% of survivors in the long term [6]. Overall, these neuropsychological postarrest challenges complicate participation in society and are associated with social issues such as failure to resume social activities and impaired RTW [3, 6, 7]. A Danish nationwide cohort study by Jørgensen and colleagues investigated RTW and the subsequent detachment from employment in cardiac populations [8]. The authors found high probabilities of not returning to work, in patients diagnosed with heart failure. These results are in line with our previous findings [2]. Six months after resuscitation, we found 58% of survivors on full-time sick leave or working notable less (10 h/week) compared to prior to the cardiac arrest. In addition, an extensive part of those who had RTW had changed job descriptions or had been assigned less demanding job tasks. Other resuscitation research support these findings, as they found that flexible work hours and/or modified job descriptions were offered to 74% of participants in a register-based OHCA population [9].

Still, the effectiveness of rehabilitation interventions for OHCA survivors are sparsely investigated [10]. Only three randomized controlled studies (393 participants) have been reported; however, none of these reported data on return-to-work. Previously, our research group has evaluated the activity of daily living (ADL) ability of 61 OHCA survivors at discharge from hospital and found that the majority had a rehabilitation potential [11]. In another study, we found that although survivors of OHCA received a variety of postarrest rehabilitation interventions, 45% still reported unmet rehabilitation needs 6 months after their cardiac arrest [2]. This is in line with qualitative findings which emphasize that especially younger cardiac arrest survivors need special attention, as they struggle against impairments and ignore them for a long time in an effort of returning to their previous daily life [12]. Hence, there is a need to develop and test a pragmatic individual-targeted intervention to facilitate RTW in survivors of OHCA.

### Objectives

The overall aim of the ROCK trial is to evaluate the effectiveness of a comprehensive individually tailored multidisciplinary rehabilitation intervention for survivors of OHCA on RTW compared to usual care. We hypothesize that the intervention will result in a higher-level labour marked attachment 1 year after hospital discharge in addition to increased health-related quality of life.

## Methods

### Trial design

The ROCK trial is a two-arm parallel group multicentre investigator-initiated pragmatic randomized controlled superiority trial with primary endpoint measured 12 months after the cardiac arrest.

### Trial sites and recruitment

Cardiac arrest survivors will be recruited from two cardiac arrest centres in the Capital Region of Denmark: Department of Cardiology, Copenhagen University Hospital-Rigshospitalet, and Department of Cardiology, Copenhagen University Hospital-Herlev and Gentofte Hospital.

### Eligibility criteria

All adult survivors with first time out-of-hospital cardiac arrest who are discharged from the hospitals will be assessed eligible for inclusion. Survivors who, prior to the cardiac arrest, were part of the labour force with at least 2 years until they are qualified to receive retirement state pensions and who can understand and fulfil the study surveys (in Danish) are eligible for inclusion. Excluded are patients with short witnessed cardiac arrests with return of spontaneous circulation estimated less than 4 min and immediate awakening without ICU treatment.

### Intervention

Individuals allocated to the intervention group will in addition to the usual care group receive a comprehensive tailored rehabilitation intervention focusing on supporting RTW.

**Fig. 1:**
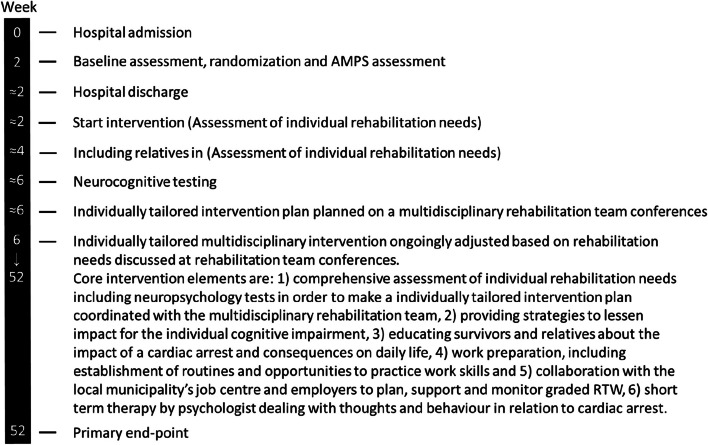
Core elements and reference activities in the intervention. In addition to the usual care, the pragmatic individual tailored rehabilitation intervention can be delivered for up to 52 weeks

The intervention is based on a multidisciplinary rehabilitation team approach (psychologists, physiotherapists, social workers, physicians within social medicine and occupational medicine, and psychiatrists). A comprehensive neurocognitive assessment and a thorough individual assessment of rehabilitation needs will be conducted to inform the individually tailored intervention plan. The plan will be developed, discussed, and planned at multidisciplinary rehabilitation team conferences. Ongoing monthly evaluation and possible adjustments of the rehabilitation plan are further discussed at multidisciplinary rehabilitation team conferences. The intervention is designed as a pragmatic individually tailored rehabilitation intervention; hence, the content and the delivery of the rehabilitation will not be uniform but based on individual needs. Therefore, based on the individual rehabilitation plan, a participant can be offered interventions that target the individual needs without limitations in relation to frequency and intensity but with a limit in relation to duration (up until 12 months after the cardiac arrest). Core intervention elements are as follows: (1) comprehensive assessment of individual rehabilitation needs including neuropsychology tests in order to make an individually tailored intervention plan coordinated with the multidisciplinary rehabilitation team; (2) providing strategies to lessen impact for the individual cognitive impairment; (3) educating survivors and relatives about the impact of a cardiac arrest and consequences on daily life; (4) work preparation, including establishment of routines and opportunities to practice work skills; (5) collaboration with the local municipality’s job centre and employers to plan, support, and monitor graded RTW; and (6) short-term therapy by psychologist dealing with thoughts and behaviour in relation to cardiac arrest. Core elements and reference activities in the intervention related to timing are presented in Fig. 1.

The complex pragmatic intervention is based on a multidisciplinary rehabilitation team approach and developed from a bio-psychosocial model perspective. The intervention is based on the principles from the individual placement and support [13] and stepped care [14] graded exercise therapy [15, 16] and cognitive behavioural therapy [17]. Individual placement and support is a model within rehabilitation focusing on RTW, where the goal is to provide professional services to help people with disabilities participate in the competitive labour market [18]. Stepped care is a system used to monitor and deliver the most effective treatment with the lease resources [14]. Graded exercise therapy is a behavioural treatment programme characterized by graded activity, time contingency, and operant conditioning. The primary aim of this rehabilitation programme is to increase the survivors’ ability to perform their own preferred activities during daily life at home or at work [15, 16]. Cognitive behavioural therapy is a short-term, goal-oriented psychotherapy treatment that takes a hands-on, practical approach to problem-solving [19].

### Usual care

All participants including those allocated to the usual care group will be seen by an occupational therapist if their MoCA screening score ≤ 26. Furthermore, if considered relevant by the discharging unit, survivors are referred for rehabilitation provided and delivered in the local municipality where the participant is resident. The content of the rehabilitation will typically be based on the content of the rehabilitation plan from the discharging hospital unit and an individual assessment of the survivors’ expressed needs, within the local municipality where the participant is resident.

### Feasibility

Each year, around 200 OHCA survivors are discharged from Copenhagen University Hospital-Rigshospitalet and Copenhagen University Hospital-Herlev & Gentofte hospitals. Approximately, 40% of these are part of the labour marked. To our knowledge, this present study will be the first randomized trial worldwide that investigates the effectiveness of a complex multidisciplinary individual tailored rehabilitation intervention targeted RTW in an OHCA population [10]. The feasibility of the intervention was tested on 10 participants. The intervention was found to be relevant and acceptable, as it was feasible to recruit and retain participants despite the COVID-19 pandemic (unpublished data). A few changes have been made to the intervention based on the feasibility testing, for example all participants allocated to the intervention will now have a comprehensive neurocognitive assessment and a thorough individual assessment of rehabilitation needs before the rehabilitation plan is discussed by the multidisciplinary rehabilitation team. Furthermore, to support the patients, the relatives can, if relevant, be offered psychological consultations either alone or with the patients.

### Outcomes

#### Primary outcome

Data for the primary outcome will be obtained from the national register on social transfer payments (DREAM) [20]. The DREAM register is a prospective register based on data from the Ministry of Employment, the Danish Education Registers, the Danish Civil Registration System (CPR registry), and the Danish Tax Authority. The register contains all citizens in Denmark, who have received one or more social transfer payments in the period from 1991 until today. The type of transfer payment is registered on a weekly basis, and the register is updated monthly.

The primary outcome of the clinical trial is labour market participation, defined as a dichotomized outcome, employment vs. on social transfer payment 12 months after hospital discharge.

This measure has been proposed as adequate and relevant measure for assessing employment prognosis following intervention on a similar group of patients [21]. Furthermore, data will be obtained by questionnaire on the degree of workload and comparable work tasks compared to pre-cardiac arrest. Hence, labour market participation 12 months after hospital discharge (employment vs. social transfer benefits (DREAM)) and labour market participation during the 12 months after hospital discharge (DREAM) will be analysed.

Sequence analysis and a multistate model analysis will be used to visualize how survivors change states during the follow-up time and to assess influences from covariates on transitions between stages.

### Secondary outcomes

All secondary outcomes: readiness for return-to-work (RRTW), Multidimensional Fatigue Inventory (MFI-20), The 5-level EuroQol 5-domain (EQ-5D-5L) quality-of-life questionnaire, European quality-of-life survey (HeartQoL), the Pittsburgh Sleep Quality Index (PSQI), Health Literacy Questionnaire (HLQ), and Impact of Event Scale-Revised (IES-R), modified job descriptions and job task, and adverse events will be collected within the first 12 weeks after hospital discharge. There will be at least one interim test at 26 weeks and again at the primary end point at 52 weeks. Please see assessment timeline in Table 1.

**Table 1:**
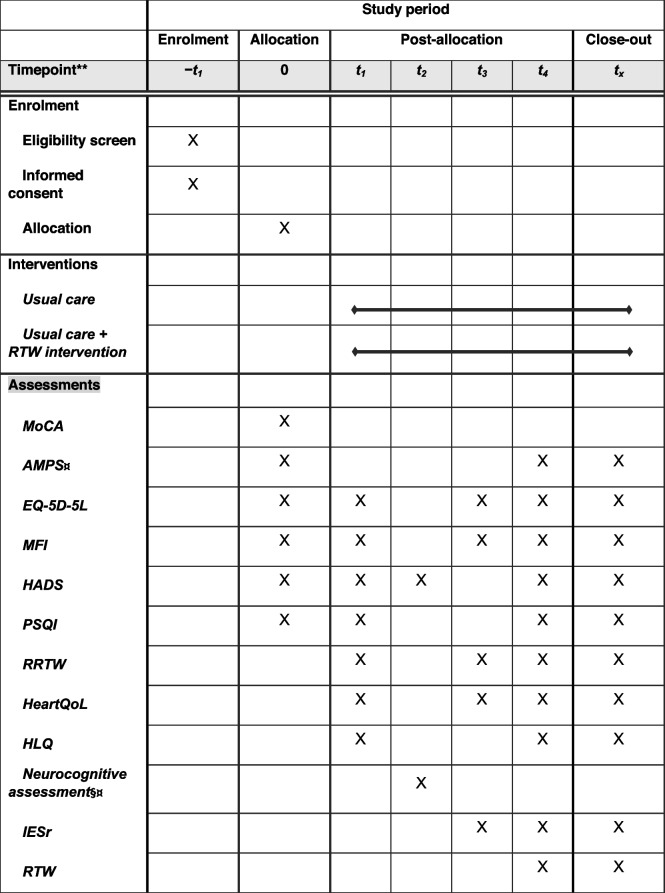
SPIRIT figure. Schedule of enrolment, allocation, interventions, and assessments

*t*_1_ 2 weeks, *t*_2_ 6 weeks, *t*_3_ 12 weeks, *t*_4_ 26 weeks, *t*_x_ 52 weeks (primary endpoint). Readiness for return to work (*RRTW*), Multidimensional Fatigue Inventory (*MFI-20*), the 5-level EuroQol-5 domain (EQ-5D-5L) quality-of-life questionnaire, European quality-of-life survey (HeartQoL), the Pittsburgh Sleep Quality Index (PSQI), Health Literacy Questionnaire (HLQ), and Impact of Event Scale-Revised (IES-R). Intervention group only. Sequence analysis of labour market trajectories using data registered in the DREAM database. Only participants allocated to the intervention, §¤list of tests; Trail making A, Trail Making B, digit span forwards, digit span backwards, RBANS list learning, RBANS story memory, Rey’s complex figure test — copy, coding, Rey’s complex figure test, immediate recall, letter number sequencing, design fluency, RBANS list recall, RBANS list recognition, RBANS memory recall, block design, verbal fluency Test (animals), verbal fluency test (S-words)

#### Readiness for return-to-work

This 22-item scale assesses the stage of readiness for return-to-work. Confirmatory factor analyses had satisfactory fit indices to confirm the initial model [22]. The scales form six subscales of precontemplation (three items) and contemplation (three items), prepared for action self-evaluative (four items), and prepared for action behaviour (three items) for participants on full-time sick leave and the stages of uncertain (five items) and proactive maintenance (four items) for participants who are working part time or full time.

#### Multidimensional fatigue inventory

Fatigue is a very commonly reported symptom in sudden cardiac arrest survivors. To measure the level of fatigue, and change over time, the 20-item patient-reported outcome measure MFI-20 was chosen. MFI-20 consist of five fatigue scales covering the following: general fatigue, physical fatigue, reduced activity, reduced motivation, and mental fatigue [23].

#### The 5-level EuroQol-5 domain

The EQ-5D-5L is a generic Health-Related quality-of-life (HRQoL) measurement tool developed as a generic instrument for HRQoL [24, 25]. EQ-5D-5L comprises five dimensions (mobility, self-care, usual activities, pain/discomfort, and anxiety/depression), each of which has five levels (no problems, slight problems, moderate problems, severe problems, or unable to) and a visual analogue scale (EQ VAS) [25]. The EQ VAS records the patient’s self-rated health on a vertical visual analogue scale, where the endpoints are labelled ‘The best health you can imagine’ and ‘The worst health you can imagine’. The EQ-5D-5L has been adapted for Danish [26], but no psychometric evaluation of the Danish version has been published. However, several studies have evaluated the English version and found acceptable psychometric properties for convergent validity and test-retest reliability [27, 28].

#### European quality-of-life survey

To measure disease-specific health-related quality of life, we have chosen the HeartQoL, which development was supported from the European Society of Cardiology and the European Association of Preventive Cardiology [29]. The 14 items patient-reported outcome has been translated into Danish and showed acceptable psychometric properties in cardiac patients following heart valve surgery, ischemic heart disease, and in recipients of implantable cardioverter defibrillator [30–32].

#### The Pittsburgh sleep quality index

PSQI is a self-reported outcome measure that assesses sleep quality and usual sleep habits. The recall frame is 1 month, and the outcome measure consists of 24 individual items measuring seven dimensions and categorized into sleep efficiency factors (sleep quality, sleep latency, sleep duration, and habitual sleep efficiency) and sleep disturbance factors (sleep disturbance, use of sleep medications, and daytime disturbance) [33].

#### Health literacy questionnaire

The Health Literacy Questionnaire (HLQ) has nine scales that each measure an aspect of the multidimensional construct of health literacy [34]. Low health literacy has been reported to be associated with increased mortality, hospitalization, lower use of preventive healthcare services, difficulty communicating with health professionals, and poorer knowledge about disease processes and self-management skills among people with chronic conditions such as heart disease. The HLQ is used in many settings, including the development of interventions, and for evaluation of health programmes [35–38].

#### Impact of event scale-revised

The IES-R is a 22-item patient-reported outcome measure that assesses subjective distress caused by traumatic events [39]. The scale is rated on a 0 (not at all) to 4 (extremely) scale with respect to how distressing each item has been during the past week. Three subscales are formed and reflect intrusion (eight items), avoidance (eight items), and hyperarousal (six items).

#### Adverse events

During the trial period, serious adverse events, adverse events, and adherence will be registered. A serious adverse event is defined as an event that leads to either death, hospitalization, or a serious risk of deterioration in health, and all other reported adverse events such as pain, fatigue, and oedema are defined as non-serious adverse. Harms will be evaluated by calculating the relative risk (RR), separately for serious and nonserious adverse event between the intervention and control group.

### Exploratory outcomes

The intervention guiding assessments MoCA [40], AMPS [41], and all neurocognitive assessments will be explored to describe rehabilitation needs at group level, knowledge that could guide clinicians and guide future development of interventions (see assessment timeline). Patient-reported data will be used to investigate the proportion of participants reporting modified job descriptions and job tasks.

### Strategies to improve adherence

The feasibility of the intervention has been tested and the intervention refined based on the gained experiences. As the study is a pragmatic study based on an individual assessment of the survivors’ expressed needs, the intervention is expected to be relevant for all participants. However, due to the single centre set-up transport to and from the study site could be a limiting factor to adherence. However, financial compensation for transport costs was offered to the participants. Furthermore, the intervention is designed to include relatives, and hence, the acceptability is expected to be relatively high, and relatives are therefore expected to be supportive and thereby increase the possibility of higher adherence to the intervention.

### Relevant concomitant care and interventions

Participants should continue to take any described medications. Irrespective of treatment allocation, participants should continue to participate in usual care activities, such as follow-up visits at the hospital or consultations with social workers. There are no restrictions regarding concomitant care during the trial.

### Criteria for discontinuing the trial

No criteria for discontinuing or modifying allocated interventions were imposed, but the survivor or their relatives could drop out of the intervention or withdraw their informed consent to participate in the study at any time, without consequences for the current or future treatment or specific for the allocated rehabilitation intervention. Should participants decide to withdraw from the trial, they will be asked if they agree that collected data, and explicit specific non-collected data form the registries, may be used for analysis.

### Recruitment, assignment of interventions, sequence generation, and allocation concealment

**Fig. 2:**
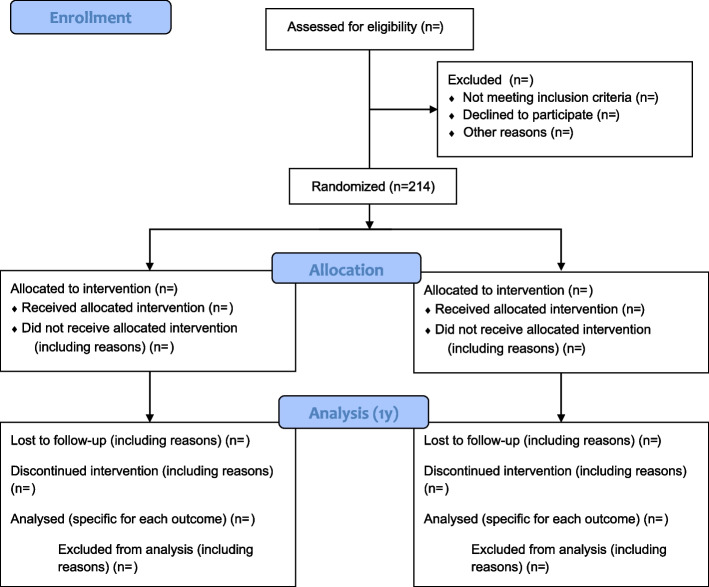
Study flow diagram

We will recruit trial participants directly from the cardiology ward. A study nurse at the ward will approach eligible survivors and invite them to participate in the present study. Participants will be randomly assigned to either the intervention or the usual care group using a computer random generator, with a 1:1 allocation by using varying block sizes. The randomisation will be conducted using the RedCap randomization module and will be stratified on sex (M/F) and age. Allocation concealment will be ensured as the randomization module block sizes are unknown, and the allocation will be locked after randomization. Clinical staff at the departments will obtain informed consent and enroll the participants. A named study author (J. C., M. K. W., or B. G. W.) will randomize participants, and the clinical staff at the departments will inform the participants about the allocation and start the intervention based on standard operating procedures. The CONSORT flow diagram is presented in Fig. 2.

### Blinding

Due to the nature of the intervention, neither participants, outcome assessors, nor staff conducting the intervention can be blinded to allocation. Group allocation will be anonymized before the data will be analysed by the data analysis responsible investigator to ensure blinding.

### Data management

All data will be handled and stored electronically in REDCap. All patient-reported and assessor-administered data entry will have checks for data values. Furthermore, all data points from assessor administered data will be cross-checked. Before extraction, all data will be anonymized, and group allocation will be blinded as group X and Y. Data will be enriched with DREAM data on labour market participation from the National Registry.

#### Statistical methods

A full statistical analysis plan will be uploaded prior to analysis at ClinicalTrials.gov (NCT05173740).

### Superiority margins

Superiority margin is set at 10%. Superiority will be claimed if confidence intervals are above 1, and a difference of 10% or more between the intervention group and the control group is detected.

### Sample size

The sample size calculation for the present trial is based on a study by Moulaert et al. who evaluated the effectiveness of a rehabilitation intervention on health-related quality of life for OHCA survivors but reported exploratory finding for RTW after 1 year. In that study, 71% of the control group had returned to work after 1 year. We expect the intervention to increase this by 25% corresponding to a RTW rate of 88.8%. Using the formula for large-sample tests for proportions in a two-sample parallel design with a superiority margin of 10% and a power of 80%, 85 survivors are needed in each arm, 170 in total. Based on the sample size calculation and an expected 20% dropout rate, 107 OHCA survivors are needed in each study arm, 214 in total [42].

### Primary outcome

The primary outcome will be analysed using logistic regression assessing RTW status at 12 months adjusting for the intervention and age at OHCA, sex, marital status, and occupation prior to OHCA. Patient-reported data on return-to-work will be used to enrich the analysis (e.g. self-employed persons, activation of substantial self-regulated pensions, unemployed without social transfer payment).

Sequence analysis will visualize how survivors change states during the follow-up time [43, 44] but also describe time spent in each state (working vs. social transfer benefit, etc.) and order of states during follow-up. However, sequence analysis is exploratory and cannot answer hypotheses or be adjusted for covariates.

Because return to work is complex, transitions between work states will be explored further. Multistate models can be used to assess influences from covariates on transitions between stages, e.g. from working to social transfer benefit, or reverse, or from working until death.

Retirement is an important state in this setting; however, retirement to private pensions is not registered in the DREAM database [20]. Thus, a multistate model including this important state will have to be built using data from survivors’ questionnaires. This is possible, as retirement is an absorbing state see (Supplemental Figure 1).

### Secondary outcomes

Secondary outcomes will be analysed using generalized linear mixed-effects models. Data are measured repeatedly, but outcomes are binary or on ordinal scale. Crude and adjusted analysis will be presented. Covariates that will be adjusted for include age at OHCA, sex, educational level, and marital status. Continuous data will be analysed using *t*-test for normally distributed data and Wilcoxon’s signed-rank test for non-normally distributed data. For categorical data, chi-square test will be used.

### Methods used for assumptions to be checked for statistical methods

In logistic regression, the linearity of log odds for each quantitative variable will be assessed by residual plots. Cooks distance and dfbeta values will be used to assess influential observations. In multistate models, the Markov assumption (previous states do not influence next transition) will be tested using the log-rank-based test [45].

The assumption of time homogeneity (that time spent in a state does not influence next transition) will be assessed using Schoenfeld residuals and by fitting a time homogeneity model and a piecewise constant model and comparing these using a likelihood ratio test. Repeated measures to identify the optimal covariance structure, models with compound symmetry, unstructured covariance, and auto-regressive with heterogeneous variance will be built and compared with ANOVA. The model with the lowest AIC and smallest −2 log likelihood score will be fitted as the final model. Time points should be equally spaced, as survivors fill surveys at approximate times during the study period. Normality will be assessed with a histogram of the residuals. Homoscedasticity will be assessed by plotting fitted values vs. residuals. Influential data points are not likely, as values are defined by surveys. The final model will be adjusted for values at baseline, to take individual variation into account.

### Details of alternative methods to be used if distributional assumptions do not hold

In logistic regression, non-linear variables will be transformed or categorized to fulfil the assumption of linearity with log odds. In multistate models, if the Markov assumption is not fulfilled, a semi-Markov model (clock reset model) will be assessed, and time of entry into a previous or current state will be included as a time-dependent covariate (state arrival Markov model). If time homogeneity is not found, a piecewise constant model will be fitted. For repeated measures, non-normality or lack of homoscedasticity will be handled by log-transformation or categorizing variables or outcome.

### Adherence to the intervention

Adherence to the allocated intervention is defined as survivors having completed the thorough individual assessment of rehabilitation needs, having completed the neurocognitive assessment, and being informed of the individually tailored intervention plan, regardless of a potential drop-out from the intervention after these core intervention elements.

### Per protocol population

For survivors to be included in the per protocol analysis on the primary outcome, they should be classified as adherent to the intervention. Furthermore, to be included in the per protocol analysis for the secondary outcome, the first assessment timepoint and an assessment after 52 weeks are needed. No exclusion criteria will be imposed in relation to the usual care group.

### Protocol deviations

Deviations from this protocol will be recorded and disseminated as protocol deviations with classification of risk (minor/major).

### Missing data and robustness

Missing data will be investigated by producing tables that characterize survivors with missing data vs. survivors with information for each missing variable, as outlined in Supplemental Table 1. There may be missing data in many variables, so variables of interest will be assessed.

If survivors with missing data differ from survivors without missing data, it is assumed that data is missing not at random and multiple imputation may be redundant. Otherwise, multiple imputation may be used as a sensitivity analysis. Whether imputation can be used will be based on a judgment of the extent of patterns in missingness. All secondary outcomes will be assessed as cross tables with intervention, primary outcome, age, sex, marital status, and occupation. As a rule of thumb, differences should be less than 5% between survivors with and without missing data, but the total pattern will be considered.

### Definition of analysis

Intention to treat, complete cases, and per protocol will be conducted for primary and secondary outcomes. Intention to treat is the sample of all survivors included who attended their first visit at the Center of Social Medicine. Complete case is the population with either a baseline assessment or assessment after 2 weeks and either a complete 6-month or 12-month assessment, for secondary end points to be derived. The per protocol population is the population of survivors with a rehabilitation plan including planned RTW interventions.

Sensitivity analysis will be conducted for the primary outcome on the following subgroups: blue collar vs. white collar, manual labour vs. non-manual, and doing several tasks at the same time vs. work concentrated on one issue at a time.

### Data monitoring

All data will be handled and stored electronically in REDCap. JC, BGW, and MKW will function as an internal data monitoring committee and will monthly discuss progress and safety data. The Regional Ethics Committee and the Danish Data Protection Agency are obliged by law to conduct random inspections of all ongoing studies. Before extraction, all data will be anonymized, and group allocation will be blinded as groups X and Y. Data will be enriched with DREAM data on labour market participation at SMC, and extracted data will be stored in a secure folder. No stopping guidelines have been defined.

### Planned reporting

Participant’s characteristics are planned to be reported as outlined in Supplemental Table 2. The primary outcome will be reported as unadjusted and adjusted OR of RTW at 12 months (see Table 2). The multistate model will be reported as outlined in Table 5 and visualized using sequence analysis (see Table 3).

**Table 2:** Unadjusted and adjusted OR of RTW at 12 months. Odds ratio (OR), 95% confidence interval (95% CI)

	Unadjusted OR RTW (95% CI)	Adjusted OR RTW (95% CI)
Intervention	OR (95% CI)	OR (95% CI)
Age	OR (95% CI)	OR (95% CI)
Male sex	OR (95% CI)	OR (95% CI)
Marital status	OR (95% CI)	OR (95% CI)
Occupation	OR (95% CI)	OR (95% CI)

**Table 3:** Unadjusted and adjusted hazard ratio (HR) of RTW at 12 months. 95% confidence interval (95% CI). May include estimates from transient intensity matrix/HR from multistate models

	Unadjusted HR sick leave: full time (95% *CI*)	Adjusted HR sick leave: full time (95% *CI*)	Unadjusted HR sick leave: part time (95% *CI*)	Adjusted HR sick leave: part time (95% *CI*)
Intervention	HR (95% CI)	HR (95% CI)	HR (95% CI)	HR (95% CI)
Age	HR (95% CI)	HR (95% CI)	HR (95% CI)	HR (95% CI)
Sex	HR (95% CI)	HR (95% CI)	HR (95% CI)	HR (95% CI)
Marital status	HR (95% CI)	HR (95% CI)	HR (95% CI)	HR (95% CI)
Occupation	HR (95% CI)	HR (95% CI)	HR (95% CI)	HR (95% CI)
	Unadjusted HR part time: retired (95% *CI*)	Adjusted HR part time: retired (95% *CI*)	Unadjusted HR full time: retired (95% *CI*)	Adjusted HR full time: retired (95% *CI*)
Intervention	HR (95% CI)	HR (95% CI)	HR (95% CI)	HR (95% CI)
Age	HR (95% CI)	HR (95% CI)	HR (95% CI)	HR (95% CI)
Sex	HR (95% CI)	HR (95% CI)	HR (95% CI)	HR (95% CI)
Marital status	HR (95% CI)	HR (95% CI)	HR (95% CI)	HR (95% CI)
Occupation	HR (95% CI)	HR (95% CI)	HR (95% CI)	HR (95% CI)

Unadjusted and adjusted estimates for secondary end points will be reported as outlined in Supplemental Table 3. RTW at 6 months will be assessed with logistic regression, other variables with generalised linear mixed models. Results for PROMs will be reported for each subscale separately in text.

Furthermore, patient-reported RTW, the degree of RTW in hours weekly compared to prior to the cardiac arrest), changed job description, and modification in job task will be reported descriptively.

### Ethics and dissemination

The study will comply with the ethical principles for medical research as described in the Declaration of Helsinki II, and the study has been approved by the ethics committee (j.nr. H-20049654).

Survivors eligible for inclusion will receive oral and written information about the study before signing an informed consent to participate. If the survivors agree to participate, they will be asked to provide written informed consent in consultation with their closest relative prior to inclusion. This is justified in ethical issues as some survivors with cognitive impairments may not feel empowered to refuse participation. All participants will be informed that all personal information is confidential, and that they have access to personal documents and records according to current legislation. Furthermore, all participants will be informed that participation is voluntary, and that they have the right to withdraw from the trial without explanation and without consequences for their future treatment. If a trial participant is excluded from the trial, either based on the participant’s own choice or the treating physician’s judgment, the participant will be asked for permission to use already collected data.

### Protocol amendments

Any modifications or amendment to the protocol or the statistical analysis plan after this study protocol has been published will be registered and justified in the ClinicalTrials.gov registration (NCT05173740).

### Confidentiality

All data obtained during the trial will be handled and stored as approved by the Danish Data Protection Agency (P-2021-393).

### Availability of data and materials

Data can be available from the corresponding author on reasonable request.

### Dissemination of results

Both negative, inconclusive, and positive findings from the trial will be disseminated at regional, national, and international conferences and in internationally high-level and disease-specific peer-reviewed journals. Dissemination to relevant stakeholders, decision-makers, survivors, relatives, and to the public in general will be planned with the research partner (Hjerneskadeforeningen) and might be coordinated with the Danish Heart Foundation, if relevant. Funders of the present study will be informed about the dissemination but will have no active role in in the dissemination of the results.

### Trial registration and status

Handling of data has been approved by the Danish Data Protection Agency (P-2021-393), and the study has been registered at ClinicalTrials.gov (NCT05173740). Recruitment started February 1, 2022, and the last visit for the last patient is anticipated to be December 31, 2026.

### Sources of monetary, material, and other support

This project has received funding from the Danish Health Foundation, Axel Muusfeldts Foundation, and the Capital Region of Denmark Research Fund. This funding sources had no role in the design of this study and will not have any role during its execution, analyses, interpretation of the data, or decision to submit results. The Danish patient organization for patients and relatives with acquired brain damage (Hjerneskadeforeningen) is official research partner and has actively contributed to discussions regarding the content of the intervention and complementary study ideas, and dissemination of results including academic, public, and relevant stakeholder dissemination will be planned as a part of the partnership. The project sponsor is the Clinical Research Unit at Department of Cardiology at Copenhagen University Hospital-Rigshospitalet. The sponsor played no role in the design of the study but approved the study. The sponsor regularly reviews project status reports from the project manager.

### Supplementary Information

**Additional file 1:** **Supplemental Figure 1.** Multi-state model. Multi-state model with 3 transient states and 1 absorbing state. As very few survivors may die during follow-up, this level may be superfluous. Death is therefore excluded in this model and retirement is the only absorbing state. However, retirement must be collected from the survivors, as it is not present in the DREAM database.

** Additional file 2:** **Supplemental Table 1.** Missing data. Standard deviation (SD). A blue-collar worker is a person who performs manual labor. Survivors who completed the thorough individual assessment of rehabilitation needs and completed the neurocognitive assessment, and who were informed of the individually tailored intervention plan is considered adherent to the intervention.

** Additional file 3:** **Supplemental Table 2.** Participant characteristics. Body mass index (BMI), A blue-collar worker is a person who performs manual labor. New York Heart Association (NYHA) Classification. Return of spontaneous circulation (ROSC). The Montreal Cognitive Assessment (MoCA). Hospital Anxiety and Depression Scale (HADS).

** Additional file 4:** **Supplemental Table 3.** Secondary outcomes. RTW based on sequence analysis of labour market trajectories using data registered in the DREAM database. Readiness for return-to-work (RRTW), The 5-level EuroQol-5 Domain (EQ-5D-5L) Quality of life questionnaire, European Quality of life survey (HeartQoL), Multidimensional Fatigue Inventory (MFI-20), symptoms of anxiety and depression measured using Hospital Anxiety and Depression Scale (HADS), Health literacy Questionnaire (HLQ), The Pittsburgh Sleep Quality Index (PSQI), and Impact of Event Scale - Revised (IES-R). *Global scores will be presented in the table and subscales scores will be reported in text or as supplementary material.
