# Supplementary material for: The ROCK trial—a multidisciplinary Rehabilitation intervention for sudden Out-of-hospital Cardiac arrest survivors focusing on return-to-worK: a pragmatic randomized controlled trial protocol
Source: Trials. 2024 Feb 1;25:99. doi: 10.1186/s13063-024-07911-6 (PMC10835971; doi:10.1186/s13063-024-07911-6)
Supplement: Supplementary file 1 — Additional file 1: Supplemental Figure 1. Multi-state model. Multi-state model with 3 transient states and 1 absorbing state. As very few survivors may die during follow-up, this level may be superfluous. Death is therefore excluded in this model and retirement is the only absorbing state. However, retirement must be collected from the survivors, as it is not present in the DREAM database. [file 13063_2024_7911_MOESM1_ESM.docx]

**Supplemental Figure 1**


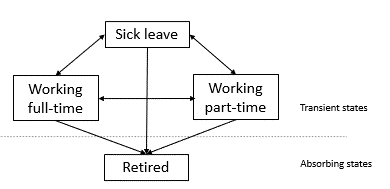


**Supplemental Figure 1: Multi-state model.** Multi-state model with 3 transient states and 1 absorbing state. As very few survivors may die during follow-up, this level may be superfluous. Death is therefore excluded in this model and retirement is the only absorbing state. However, retirement must be collected from the survivors, as it is not present in the DREAM database.
