# Supplementary material for: The ROCK trial—a multidisciplinary Rehabilitation intervention for sudden Out-of-hospital Cardiac arrest survivors focusing on return-to-worK: a pragmatic randomized controlled trial protocol
Source: Trials. 2024 Feb 1;25:99. doi: 10.1186/s13063-024-07911-6 (PMC10835971; doi:10.1186/s13063-024-07911-6)
Supplement: Supplementary file 2 — Additional file 2: Supplemental Table 1. Missing data. Standard deviation (SD). A blue-collar worker is a person who performs manual labor. Survivors who completed the thorough individual assessment of rehabilitation needs and completed the neurocognitive assessment, and who were informed of the individually tailored intervention plan is considered adherent to the intervention. [file 13063_2024_7911_MOESM2_ESM.docx]

**Supplemental Table 1**

|  | **Missing (n=xx)** | **Not missing (n=YY)** |
| --- | --- | --- |
| Primary outcome | n, % | n, % |
| Intervention, drop-out | n, % | n, % |
| Intervention, non-adherent | n, % | n, % |
| Age (years) | Mean (SD) | Mean age (SD) |
| Sex (male) | n, % | n, % |
| Marital status (Cohabiting/married) | n, % | n, % |
| Occupation (Blue-Collar) | n, % | n, % |

**Supplemental Table 1: Missing data.** Standard deviation (SD). A blue-collar worker is a person who performs manual labor. Survivors who completed the thorough individual assessment of rehabilitation needs and completed the neurocognitive assessment, and who were informed of the individually tailored intervention plan is considered adherent to the intervention.
