# Supplementary material for: The ROCK trial—a multidisciplinary Rehabilitation intervention for sudden Out-of-hospital Cardiac arrest survivors focusing on return-to-worK: a pragmatic randomized controlled trial protocol
Source: Trials. 2024 Feb 1;25:99. doi: 10.1186/s13063-024-07911-6 (PMC10835971; doi:10.1186/s13063-024-07911-6)
Supplement: Supplementary file 3 — Additional file 3: Supplemental Table 2. Participant characteristics. Body mass index (BMI), A blue-collar worker is a person who performs manual labor. New York Heart Association (NYHA) Classification. Return of spontaneous circulation (ROSC). The Montreal Cognitive Assessment (MoCA). Hospital Anxiety and Depression Scale (HADS). [file 13063_2024_7911_MOESM3_ESM.docx]

**Supplemental Table 2**

|  | Intervention (n=) | Usual care (n=) |
| --- | --- | --- |
| Age (years) | Mean (SD) | Mean (SD) |
| Sex (male) | n, % | n, % |
| BMI | Mean (SD) | Mean (SD) |
| Marital status (Cohabiting/married) | n, % | n, % |
| Occupation (Blue-Collar) | n, % | n, % |
| NYHA class (I-IV) | n, % | n, % |
| Witnessed cardiac arrest (yes) | n, % | n, % |
| Number of defibrillations | n, % | n, % |
| Time to ROSC (min) | Mean (SD) | Mean (SD) |
| Type(s) of cardiovascular disease | n, % | n, % |
| Charlson Comorbidity Index | Mean (SD) | Mean (SD) |
| MOCA | Mean (SD) | Mean (SD) |
| HADS anxiety | Mean (SD) | Mean (SD) |
| HADS depression | Mean (SD) | Mean (SD) |

**Supplemental Table 2: Participant characteristics.** Body mass index (BMI), A blue-collar worker is a person who performs manual labor. New York Heart Association (NYHA) Classification. Return of spontaneous circulation (ROSC). The Montreal Cognitive Assessment (MoCA). Hospital Anxiety and Depression Scale (HADS).
