# Supplementary material for: The ROCK trial—a multidisciplinary Rehabilitation intervention for sudden Out-of-hospital Cardiac arrest survivors focusing on return-to-worK: a pragmatic randomized controlled trial protocol
Source: Trials. 2024 Feb 1;25:99. doi: 10.1186/s13063-024-07911-6 (PMC10835971; doi:10.1186/s13063-024-07911-6)
Supplement: Supplementary file 4 — Additional file 4: Supplemental Table 3. Secondary outcomes. RTW based on sequence analysis of labour market trajectories using data registered in the DREAM database. Readiness for return-to-work (RRTW), The 5-level EuroQol-5 Domain (EQ-5D-5L) Quality of life questionnaire, European Quality of life survey (HeartQoL), Multidimensional Fatigue Inventory (MFI-20), symptoms of anxiety and depression measured using Hospital Anxiety and Depression Scale (HADS), Health literacy Questionnaire (HLQ), The Pittsburgh Sleep Quality Index (PSQI), and Impact of Event Scale - Revised (IES-R). *Global scores will be presented in the table and subscales scores will be reported in text or as supplementary material. [file 13063_2024_7911_MOESM4_ESM.docx]

**Supplemental Table 3**

|  | Unadjusted  RTW at 6 months | Adjusted  RTW at 6 months | Unadjusted  RRTW* | Adjusted  RRTW* |
| --- | --- | --- | --- | --- |
| Intervention | OR (95%CI) | OR (95%CI) | Est (95%CI) | Est (95%CI) |
| Age | OR (95%CI) | OR (95%CI) | Est (95%CI) | Est (95%CI) |
| Male sex | OR (95%CI) | OR (95%CI) | Est (95%CI) | Est (95%CI) |
| Marital status | OR (95%CI) | OR (95%CI) | Est (95%CI) | Est (95%CI) |
| Occupation | OR (95%CI) | OR (95%CI) | Est (95%CI) | Est (95%CI) |
|  | Unadjusted  EQ-5D-5L* | Adjusted  EQ-5D-5L* | Unadjusted  HeartQoL* | Adjusted  HeartQoL* |
| Intervention | Est (95%CI) | Est (95%CI) | Est (95%CI) | Est (95%CI) |
| Age | Est (95%CI) | Est (95%CI) | Est (95%CI) | Est (95%CI) |
| Male sex | Est (95%CI) | Est (95%CI) | Est (95%CI) | Est (95%CI) |
| Marital status | Est (95%CI) | Est (95%CI) | Est (95%CI) | Est (95%CI) |
| Occupation | Est (95%CI) | Est (95%CI) | Est (95%CI) | Est (95%CI) |
|  | Unadjusted  MFI-20* | Adjusted  MFI-20* | Unadjusted  anxiety | Adjusted  anxiety |
| Intervention | Est (95%CI) | Est (95%CI) | Est (95%CI) | Est (95%CI) |
| Age | Est (95%CI) | Est (95%CI) | Est (95%CI) | Est (95%CI) |
| Male sex | Est (95%CI) | Est (95%CI) | Est (95%CI) | Est (95%CI) |
| Marital status | Est (95%CI) | Est (95%CI) | Est (95%CI) | Est (95%CI) |
| Occupation | Est (95%CI) | Est (95%CI) | Est (95%CI) | Est (95%CI) |
|  | Unadjusted depression | Adjusted depression | Unadjusted  HLQ* | Adjusted  HLQ* |
| Intervention | Est (95%CI) | Est (95%CI) | Est (95%CI) | Est (95%CI) |
| Age | Est (95%CI) | Est (95%CI) | Est (95%CI) | Est (95%CI) |
| Male sex | Est (95%CI) | Est (95%CI) | Est (95%CI) | Est (95%CI) |
| Marital status | Est (95%CI) | Est (95%CI) | Est (95%CI) | Est (95%CI) |
| Occupation | Est (95%CI) | Est (95%CI) | Est (95%CI) | Est (95%CI) |
|  | Unadjusted  PSQI* | Adjusted  PSQI* | Unadjusted  IES-R* | Adjusted  IES-R* |
| Intervention | Est (95%CI) | Est (95%CI) | OR (95%CI) | OR (95%CI) |
| Age | Est (95%CI) | Est (95%CI) | OR (95%CI) | OR (95%CI) |
| Male sex | Est (95%CI) | Est (95%CI) | OR (95%CI) | OR (95%CI) |
| Marital status | Est (95%CI) | Est (95%CI) | OR (95%CI) | OR (95%CI) |
| Occupation | Est (95%CI) | Est (95%CI) | OR (95%CI) | OR (95%CI) |

**Supplemental Table 3. Secondary outcomes.** RTW based on sequence analysis of labour market trajectories using data registered in the DREAM database. Readiness for return-to-work (RRTW), The 5-level EuroQol-5 Domain (EQ-5D-5L) Quality of life questionnaire, European Quality of life survey (HeartQoL), Multidimensional Fatigue Inventory (MFI-20), symptoms of anxiety and depression measured using Hospital Anxiety and Depression Scale (HADS), Health literacy Questionnaire (HLQ), The Pittsburgh Sleep Quality Index (PSQI), and Impact of Event Scale - Revised (IES-R). *Global scores will be presented in the table and subscales scores will be reported in text or as supplementary material.
